# Effects of drying time on the formation of merged and soft MAPbI_3_ grains and their photovoltaic responses†

**DOI:** 10.1039/d2na00929c

**Published:** 2023-03-02

**Authors:** Anjali Chandel, Qi Bin Ke, Shou-En Chiang, Hsin-Ming Cheng, Sheng Hsiung Chang

**Affiliations:** a Department of Physics, Chung Yuan Christian University Taoyuan 320314 Taiwan Republic of China shchang@cycu.edu.tw; b Research Center for Semiconductor Materials and Advanced Optics Taoyuan 320314 Taiwan Republic of China; c Center for Nano Technology and R&D Center for Membrane Technology, Chung Yuan Christian University Taoyuan 320314 Taiwan Republic of China; d Department of Photonics, National Cheng Kung University Tainan 701 Taiwan Republic of China smcheng.jemmy@gmail.com

## Abstract

The grain sizes of soft CH_3_NH_3_PbI_3_ (MAPbI_3_) thin films and the atomic contact strength at the MAPbI_3_/P3CT-Na interface are manipulated by varying the drying time of the saturated MAPbI_3_ precursor solutions, which influences the device performance and lifespan of the resultant inverted perovskite photovoltaic cells. The atomic-force microscopy images, cross-sectional scanning electron microscopy images, photoluminescence spectra and absorbance spectra show that the increased short-circuit current density (*J*_SC_) and increased fill factor (FF) are mainly due to the formation of merged MAPbI_3_ grains. Besides, the open-circuit voltage (*V*_OC_) of the encapsulated photovoltaic cells largely increases from 1.01 V to 1.15 V, thereby increasing the power conversion efficiency from 17.89% to 19.55% after 30 days, which can be explained as due to the increased carrier density of the MAPbI_3_ crystalline thin film. It is noted that the use of the optimized drying time during the spin coating process results in the formation of merged MAPbI_3_ grains while keeping the contact quality at the MAPbI_3_/P3CT-Na interface, which boosts the device performance and lifespan of the resultant perovskite photovoltaic cells.

## Abstract 2

The grain sizes of MAPbI_3_ films and the atomic contact strength at the MAPbI_3_/P3CT-Na interface are manipulated by varying the drying time of saturated MAPbI_3_ precursor solutions, which influences the performance of the resultant perovskite photovoltaic cells.

## Graphical abstract

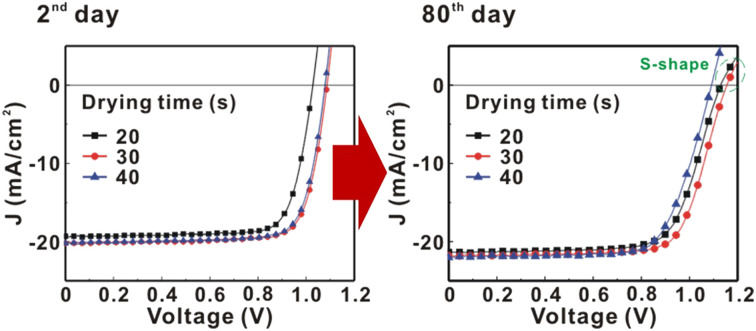

## Introduction

Solution-processed perovskite crystalline thin films have been widely used in various applications, such as photovoltaic cells,^1–3^ light-emitting diodes,^4–6^ photo-detectors,^7–9^ memory devices^10–12^ and field-effect transistors,^13–15^ mainly due to their tunable crystal structure and superior optoelectronic properties.^16–18^ Organometal trihalide perovskites are suitable for solar cells as the light absorbing layer owing to the large absorption coefficient, moderate refractive index, small exciton binding energy and long carrier diffusion length,^19–21^ which results in a high power conversion efficiency (PCE) of 25.6% (25.0%) when the regular-type (inverted-type) structure is used.^22,23^ In the lead trihalide perovskite photovoltaic cells, the size of the multi-crystalline grains was increased from several hundred nanometers to several micrometers in order to reduce the defect density at the grain boundaries of the light absorbing layer.^24–26^ However, the larger grain (lower surface area) corresponds to the lower doping concentration of the perovskite crystalline thin film, thereby resulting in the lower open-circuit voltage (*V*_OC_) of the resultant cells.^27–29^ In other words, the ideal *V*_OC_ of the intrinsic perovskite based photovoltaic cells is related to the Fermi levels of the electron transport layer (ETL) and hole transport layer (HTL).^30–32^ In recent years, the power conversion efficiency (PCE) of the perovskite photovoltaic cells can be higher than 20% when the grains of the light absorbing layer are sub-micrometer size,^33–35^ which means that the defects at the grain boundaries are effectively passivated, thereby resulting in low carrier recombination (high fill factor). In other words, there is an optimal grain size of the perovskite crystalline thin films in the highly efficient and stable perovskite photovoltaic cells. In the highly efficient and stable perovskite photovoltaic cells, the grain sizes of the light absorbing layer are ranging from 300 nm to 500 nm,^22,23,33–35^ which is slightly larger than the size of the surface feature in the ITO thin film.^36,37^ It is noted that the layered structure is a common feature of these perovskite grains,^38^ which indicates that the sub-micrometer-sized particles are single-crystalline grains.^39^ In other words, the optimized size of perovskite grains is highly related to the size of the surface feature in the ITO thin film. On the other hand, the residual solvent molecules in the solution-processed perovskite grain thin films degrade the device performance and lifespan of the resultant photovoltaic cells,^40–42^ which indicates the importance of the drying process during the formation of high-quality perovskite thin films.

In this study, the main aim is to investigate the effects of the perovskite grain size on the device performance of the P3CT-Na HTL based inverted perovskite photovoltaic cells with a facile encapsulation method.^43^ Our experimental results show that the re-dissolving process of perovskite nucleation can be used to manipulate the grain size of the perovskite crystalline thin films which strongly influences the *V*_OC_ and lifespan of the encapsulated perovskite photovoltaic cells.

## Experiments

Poly[3-(4-carboxybutyl)thiophene-2,5-diyl] (P3CT) and NaOH were purchased from Matrix Scientific and Sigma-Aldrich, respectively. The regioregularity of the used P3CT polymers is about 85%. PbI_2_ and CH_3_NH_3_I (MAI) were purchased from Uni-Onward and Lumtec, respectively. Phenyl-C_61_-butyric acid methyl ester (PCBM) and (bathocuproine) BCP were purchased from Uni-Onward and Sigma-Aldrich, respectively. Dimethylformamide (DMF), dimethyl sulfoxide (DMSO), chlorobenzene (CB) and bromobenzene (BrB) were purchased from Sigma-Aldrich. Isopropyl alcohol (IPA) was purchased from ACROS. The preparation of P3CT-Na/water solution, MAPbI_3_/DMF:DMSO solution, PCBM/CB:BrB solution and BCP/IPA solution is illustrated in our previous report.^44^ The P3CT-Na solution, perovskite precursor (1.5 M) solution, PCBM/CB:BrB solution (2 wt%) and BCP/IPA (0.089 wt%) solution were stirred at 500 rpm for 3 h at room temperature.

The device structure is Ag/BCP:PCBM/MAPbI_3_/P3CT-Na/ITO/glass. Ag and ITO are used as the cathode and anode, respectively. PCBM and P3CT-Na are used as the electron transport layer (ETL) and hole transport layer (HTL), respectively. BCP is used to modify the PCBM thin film and the contact quality at the PCBM/MAPbI_3_ interface.^45^ A MAPbI_3_ crystalline thin film is used as the light absorbing layer. The ITO/glass (7 Ω sq^−1^) substrates were modified by using a UV–ozone cleaner for 45 minutes in order to increase the surface wettability. The P3CT-Na polymer modification layer, 500 nm-thick MAPbI_3_ crystalline thin film, and 60 nm-thick PCBM thin film were fabricated by using the spin coating method. Besides, the BCP/IPA solution treatment was used to modify the PCBM thin film and the contact quality at the PCBM/MAPbI_3_ interface. The 100 nm-thick Ag thin film was fabricated by using the vacuum thermal evaporation method. The detailed information of the device fabrication process is illustrated in our previous report.^44^ The fabrication conditions of the P3CT-Na polymer ultra-thin layer, MAPbI_3_ crystalline thin film, PCBM thin film and Ag thin film are described in the ESI.† After the injection of CB into the MAPbI_3_ precursor solution, the precursor turns from a transparent solution to a light brown solution, thereby forming nucleation sites and/or intermediate states. In other words, the residual spin period can be viewed as the drying time of the MAPbI_3_ precursor solution. During the spin coating process of the MAPbI_3_ precursor solution, the spin time in the second step was changed from 29 s to 49 s in order to vary the drying times from 20 s to 40 s. The active area of a single MAPbI_3_ photovoltaic device is defined to be 2 × 5 mm^2^ by using a metallic shadow mask during the thermal evaporation process of Ag thin films. Each sample contains four cells. One glass substrate and one Parafilm were used to encapsulate the device in order to increase the device lifespan. The encapsulation method is described in Fig. S1.† The current density–voltage (*J*–*V*) curves of the inverted perovskite photovoltaic cells were obtained by using a source-meter system (NI-USB 6356 DAQ) under one sun illumination (AM1.5G, 100 mW cm^−2^). The light intensity of the light-emitting diode based solar simulator (VeraSol-2, Newport) was calibrated by using a reference cell (91150V, Newport) before the *J*–*V* curve measurement.

To investigate the trends of the *V*_OC_, *J*_SC_ and FF, MAPbI_3_/P3CT-Na/ITO/glass samples with and without encapsulation were prepared. An atomic force microscope (UTEK, Nanoview 1000) and field-emission scanning electron microscope (JEOL, JSM-7600F) were used to obtain the surface morphologies and cross-sectional images of the samples, respectively. An X-ray diffractometer (Bruker, D8 Advance) was used to characterize the crystal structures of the samples. A home-made optical spectrometer was used to measure the absorbance and reflectance spectra. The setup of the absorbance/reflectance spectrometer is illustrated in Fig. S2.† A home-made optical microscope based photoluminescence (PL) spectrometer^46^ was used to analyze the light emissions of the samples under top excitation and bottom excitation.

## Results and discussion

**Fig. 1:**
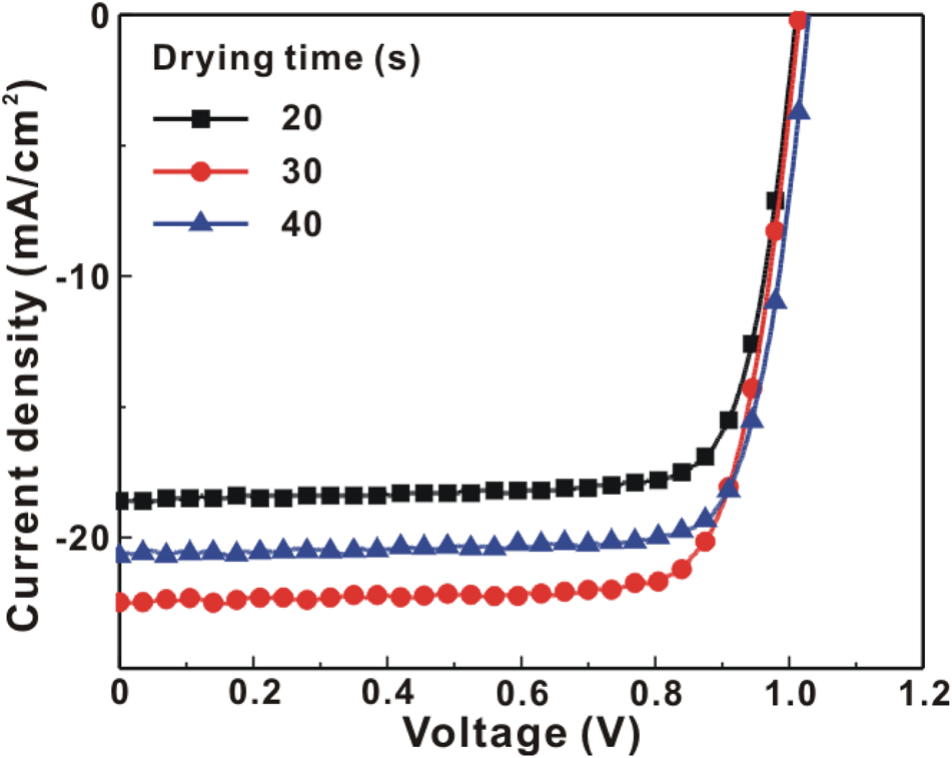
Current density–voltage (*J*–*V*) curves of the photovoltaic cells under one sun illumination (AM 1.5G, 100 mW cm^−2^). MAPbI_3_ perovskite films are prepared with different drying times.

**Fig. 2:**
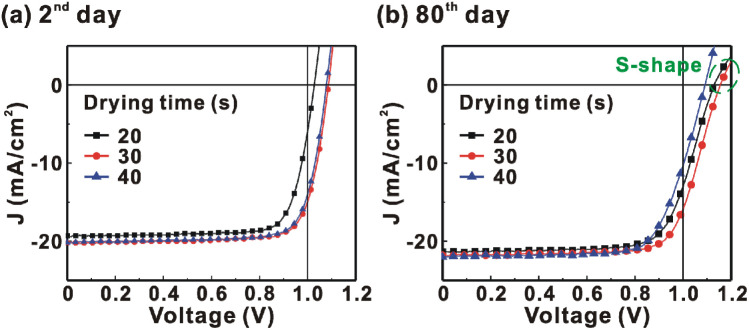
Current density–voltage (*J*–*V*) curves of the photovoltaic cells with a facile encapsulation method under one sun illumination (AM 1.5G and 100 mW cm^−2^). MAPbI_3_ perovskite films are prepared with different drying times. (a) 2^nd^ day; (b) 80^th^ day.

**Fig. 3:**
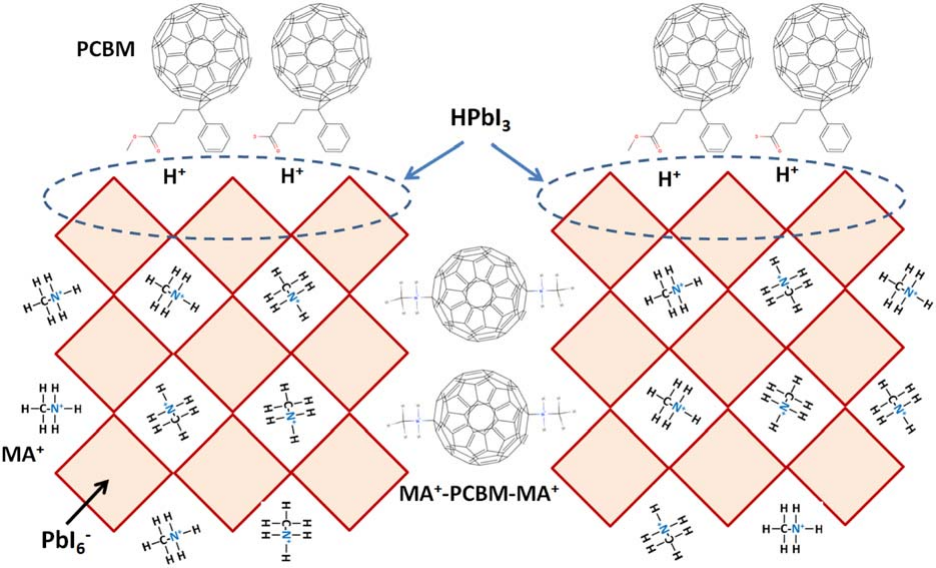
Formation of MA^+^-PCBM-MA^+^ cations and an HPbI_3_ interlayer.

**Fig. 4:**
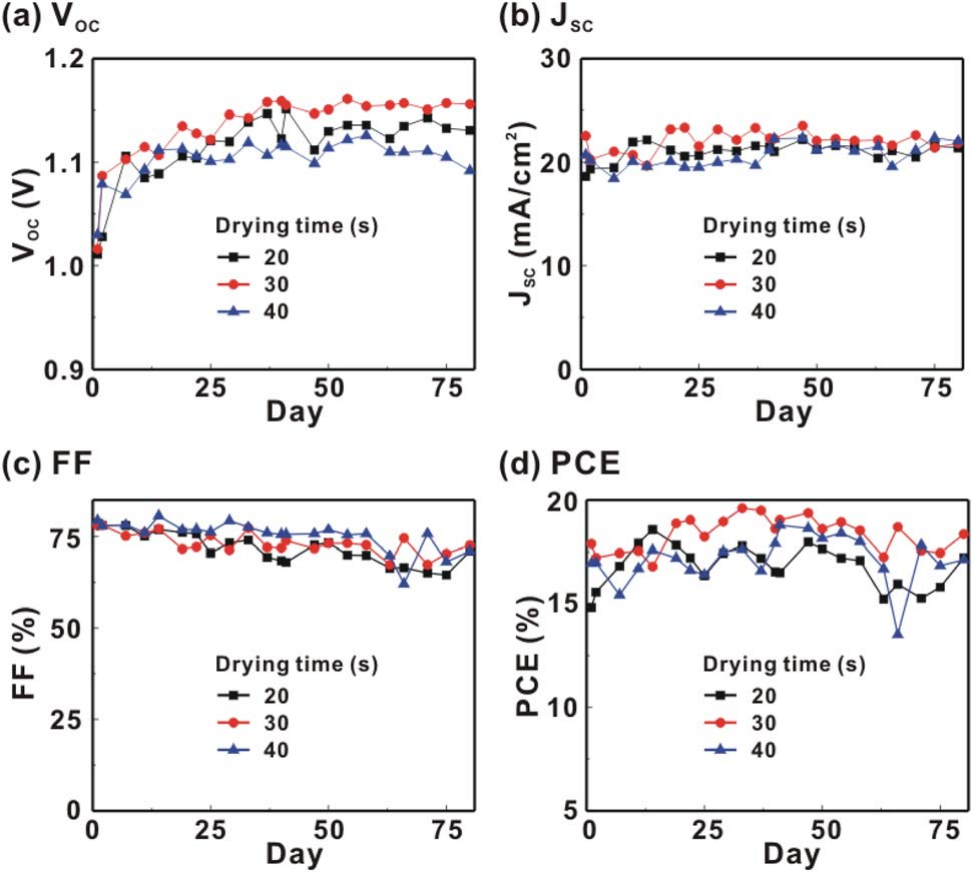
Day-dependent device performance of the photovoltaic cells under one sun illumination (AM 1.5G and 100 mW cm^−2^). MAPbI_3_ perovskite films are prepared with different drying times. (a) *V*_OC_; (b) *J*_SC_; (c) FF; (d) PCE.

Fig. 1 presents the *J*–*V* curves of the inverted perovskite photovoltaic cells under one sun illumination (AM 1.5G, 100 mW cm^−2^). The photovoltaic performance of the 8 cells for each condition is listed in Table 1. The average *V*_OC_ increases from 1.010 V to 1.017 V with the increase in the drying time from 20 s to 40 s, which means that a longer drying time results in a lower potential loss. When the drying time increases from 20 s (30 s) to 30 s (40 s), the average *J*_SC_ increases (decreases) from 19.01 mA cm^−2^ (22.25 mA cm^−2^) to 22.25 mA cm^−2^ (20.32 mA cm^−2^), which means that the drying time largely influences the contact quality at the MAPbI_3_/P3CT-Na interface, thereby dominating the collection efficiency of photo-generated holes.^47^ The photovoltaic cells were encapsulated with a facile method on the 2^nd^ day in order to increase the device lifespan.^43^ It is noted that the *V*_OC_ values increased after the encapsulation process. When the drying time is 20 s, the *J*_SC_ and FF are both increased after the encapsulation process. When the drying time is 30 s or 40 s, the *J*_SC_ and FF are both decreased after the encapsulation process, thereby slightly decreasing the PCE on the 2^nd^ day. Besides, the *V*_OC_ hysteresis of the encapsulated solar cells largely increases from 0.033 V to 0.220 V with the increase in the drying time from 30 s to 40 s (see Fig. S3†), which can be used to explain the lower *V*_OC_ and FF when the drying time is 40 s. On the 80^th^ day, the *V*_OC_ and *J*_SC_ values are both increased while maintaining the high FF values. The slightly decreased FF is mainly due to the increased series resistance (*R*_S_). Fig. 2 presents the *J*–*V* curves of the inverted perovskite photovoltaic cells on the 2^nd^ day and 80^th^ day. The *V*_OC_ and *J*_SC_ values of the encapsulated photovoltaic cells are both increased, which means that the encapsulation process increases (reduces) the exciton dissociation efficiency (potential loss). When the drying time is 20 s or 30 s, an s-shape characteristic can be observed in the *J*–*V* curves, which means that an ultra-thin potential barrier may be formed at the PCBM/MAPbI_3_ interface.^48^ In other words, it is possible to form a large-bandgap HPbI_3_ interlayer^49^ in between the PCBM and MAPbI_3_ thin films due to the formation of MA^+^-PCBM-MA^+^ cations,^50^ as shown in Fig. 3. The formation of a MA^+^-PCBM-MA^+^ cation produces two H^+^ ions *via* dehydrogenation from the two MA^+^ cations. Then, it is possible to form a HPbI_3_ interlayer when the H^+^ ions migrate to MA^+^ vacancies. It is noted that the s-shape characteristics are not observed in the *J*–*V* curves when the drying time is 40 s. Fig. 4 presents the day-dependent device performance of the best perovskite photovoltaic cells under one sun illumination. After encapsulation, the photovoltaic cells have similar trends in the day-dependent *V*_OC_, *J*_SC_ and FF. When the drying time is 30 s, the *V*_OC_ (FF) of the encapsulation photovoltaic cell largely increases (slightly decreases) from 1.087 V (78.1%) to 1.156 V (72.6%), thereby maintaining the average PCE to be higher than 17.5% within 80 days. On the 80^th^ day, the higher average PCE of the encapsulated cells is mainly due to the higher average *V*_OC_ when the drying time is 30 s. And, the differences in average *V*_OC_ values are higher than the standard deviation values of *V*_OC_ (see Table 1), which can be used to confirm that the PCE and device lifespan of the resultant perovskite photovoltaic cells are better when the drying time is 30 s. To analyze the day-dependent device performance, the *V*_OC_ curves and FF curves are fitted with an exponential growth function and a linear decay function, respectively (see Fig. S4†). The rising time values of the *V*_OC_ curves (decay rates of the FF curves) are 11.59 days (−0.0015 per day), 19.83 days (−0.0008 per day) and 18.03 days (−0.0012 per day) when the drying times are 20, 30 and 40 s, respectively. The trend of the *V*_OC_ rising time values is inversely proportional to the trend of the FF decay rate values, which is probably related to the shallow defect density in the MAPbI_3_ crystalline thin films and the formation rate of MA^+^-PCBM-MA^+^ cations and a large-bandgap HPbI_3_ interlayer at the PCBM/MAPbI_3_ interface. The existence of shallow defects does not significantly influence the exciton binding energy of perovskite crystalline thin films.^43,51^ In other words, the high *V*_OC_ of 1.156 V can be explained as due to the formation of shallow defects in the soft MAPbI_3_ crystalline thin film.^52^ To confirm the stable *J*_SC_ values, the incident photon-to-current conversion efficiency (IPCE) spectra of the encapsulated cells were measured on the 406^th^ day, as shown in Fig. S5(a).† Fig. S5(b)† shows that the integrated current density values are about 22 mA cm^−2^ which is close to the stable *J*_SC_ values. It is noted that the highest PCE is lower than 20%, which is probably due to the lower regioregularity of the used P3CT polymers.^47^

**Table 1:** Day-dependent device performance of photovoltaic cells under one sun illumination (AM 1.5G, and 100 mW cm^−2^)

Day/encapsulation	Drying time (s)	*V*_OC_ (V)	*J*_SC_ (mA cm^−2^)	FF (%)	PCE (%)	*R*_s_ (Ω)
1st/no	20	1.010 ± 0.001	19.01 ± 0.36	73.2 ± 5.3	14.05 ± 1.32	38 ± 1
1st/no	30	1.015 ± 0.001	22.25 ± 0.32	78.5 ± 0.4	17.72 ± 0.37	39 ± 1
1st/no	40	1.017 ± 0.001	20.32 ± 0.45	72.5 ± 6.5	14.98 ± 1.72	50 ± 5
2nd/with	20	1.043 ± 0.014	19.34 ± 0.04	75.1 ± 3.0	15.15 ± 0.85	58 ± 17
2nd/with	30	1.086 ± 0.001	20.00 ± 0.27	76.6 ± 1.5	16.63 ± 0.58	40 ± 1
2nd/with	40	1.045 ± 0.034	19.89 ± 0.31	70.1 ± 5.9	14.57 ± 1.99	48 ± 10
80th/with	20	1.109 ± 0.022	21.24 ± 0.17	72.8 ± 1.9	17.15 ± 0.94	98 ± 36
80th/with	30	1.133 ± 0.023	21.82 ± 0.05	71.9 ± 0.7	17.78 ± 0.57	112 ± 13
80th/with	40	1.092 ± 0.020	21.81 ± 0.26	68.5 ± 2.5	16.31 ± 1.11	152 ± 32

**Fig. 5:**
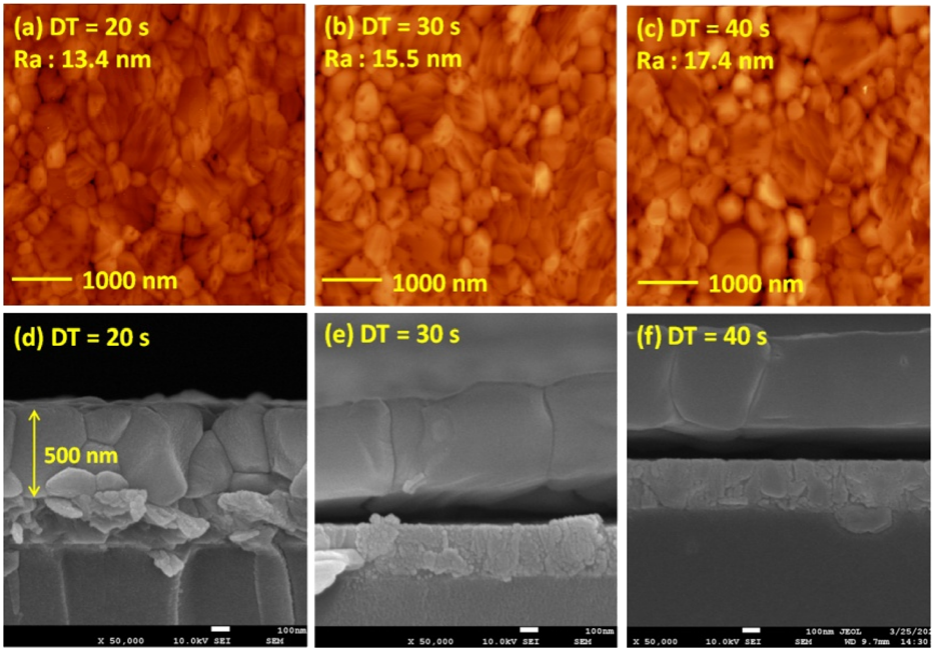
Atomic-force microscopy (AFM) images and scanning electron microscopy (SEM) images of MAPbI_3_/P3CT-Na/ITO/glass samples fabricated with different drying times (DTs). (a) AFM, DT = 20 s; (b) AFM, DT = 30 s; (c) AFM, DT = 40 s; (d) SEM, DT = 20 s; (e) SEM, DT = 30 s; (f) SEM, DT = 40 s.

**Fig. 6:**
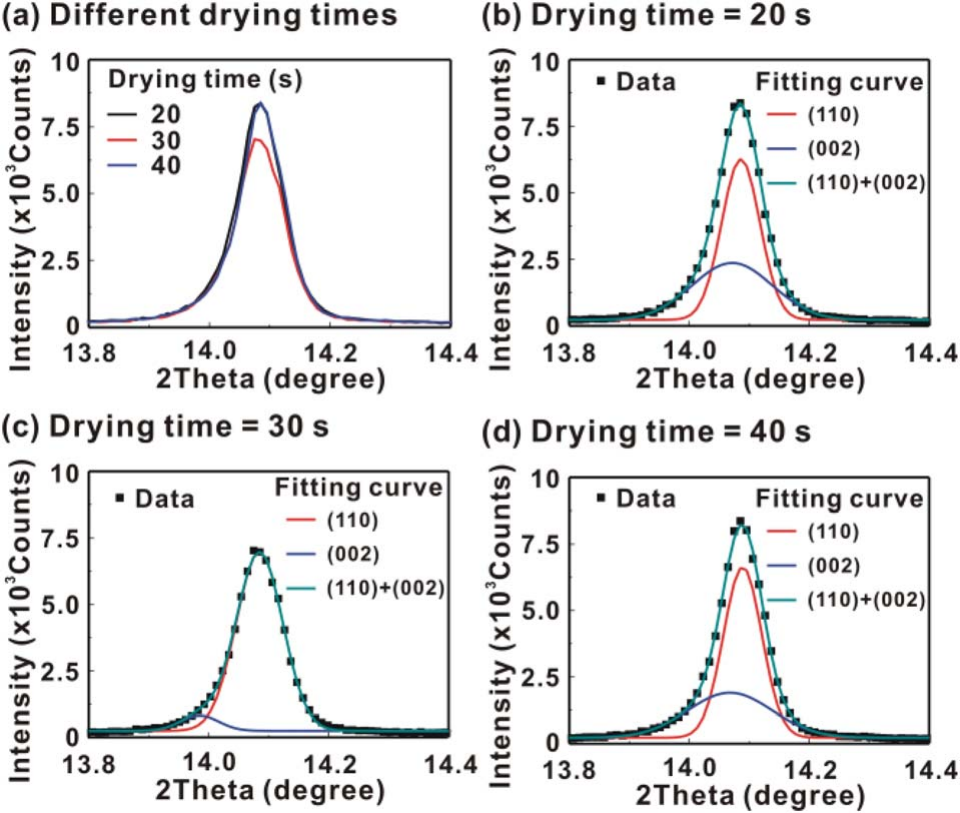
(a) Main X-ray diffraction patterns of the MAPbI_3_/P3CT-Na/ITO/glass samples fabricated with different drying times (DTs). (b) Fitting curves, DT = 20 s; (c) fitting curves, DT = 30 s; (d) fitting curves, DT = 40 s.

**Fig. 7:**
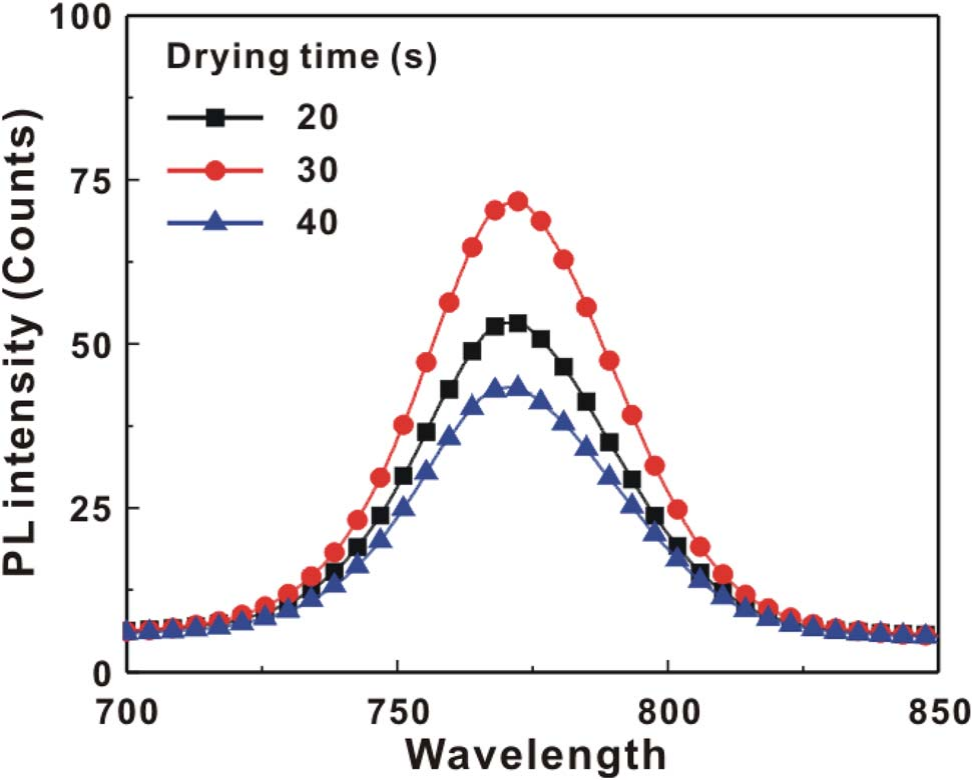
Top-excited photoluminescence spectra of the MAPbI_3_/P3CT-Na/ITO/glass samples fabricated with different drying times.

**Fig. 8:**
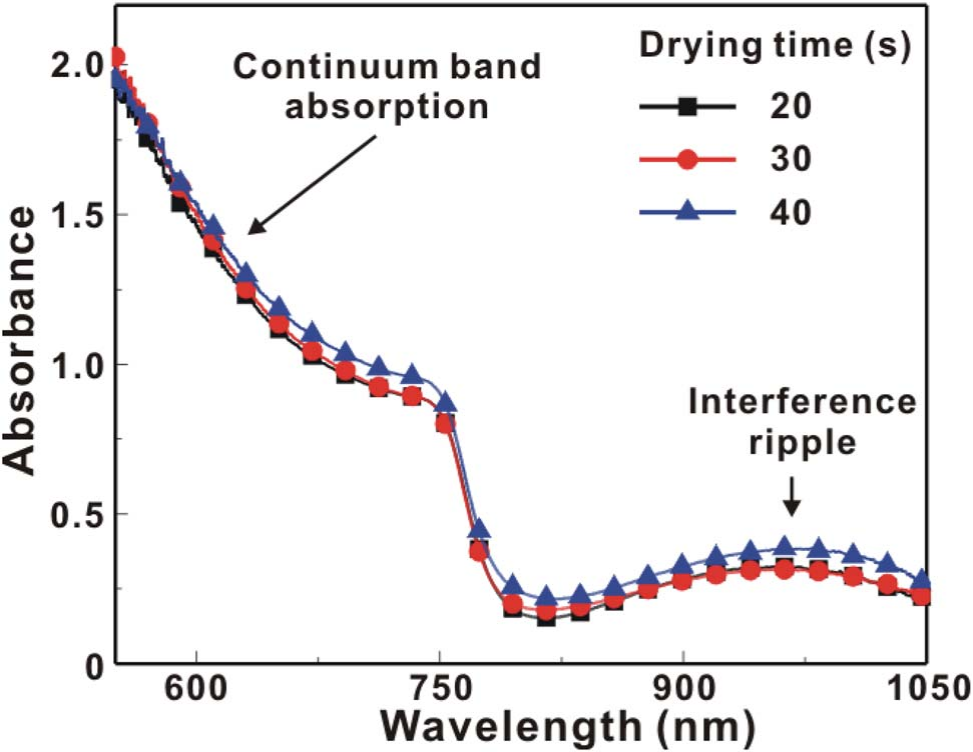
Absorbance spectra of the MAPbI_3_/P3CT-Na/ITO/glass samples fabricated with different drying times.

Fig. 5 presents the atomic force microscopy (AFM) images and cross-sectional scanning electron microscopy (SEM) images of the MAPbI_3_/P3CT-Na/ITO/glass samples fabricated with different drying times. When the drying time increases from 20 s to 40 s, the trend of the average surface roughness values (Fig. 5(a)–(c)) is proportional to the trend of the grain sizes (see Fig. 5(d)–(f)). In other words, the longer drying time results in merged MAPbI_3_ grains, thereby increasing the surface roughness which can be calculated from the AFM images. It is noted that the MAPbI_3_ crystalline thin film is separated from the substrate when the drying time is 30 s or 40 s, which means that the formation of merged MAPbI_3_ grains reduces the atomic contact strength at the MAPbI_3_/P3CT-Na interface, thereby leading to mechanical stress induced separation during the splitting process. In other words, there is a trade-off between the MAPbI_3_ grain size and the contact quality at the MAPbI_3_/P3CT-Na interface, which dominates the trends of the *J*_SC_ values and FF values of the resultant inverted perovskite photovoltaic cells on the first day (see Table 1). Besides, the trend of the grain sizes is proportional to the trend of the *V*_OC_ values and the s-shape characteristics in the *J*–*V* curve of the resultant inverted perovskite photovoltaic cells on the first day (see Table 1) due to the recued potential loss at grain boundaries.^53,54^ The trend of the grain sizes also influences the formation of s-shape characteristic in the *J*–*V* curves on the 80th day (see Fig. 2(b)), which means that the larger grains (less grain boundaries) result in less MA^+^-PCBM-MA^+^ cations, thereby reducing the formation of a HPbI_3_ barrier at the PCBM/MAPbI_3_ interface. Fig. 6(a) presents the main X-ray diffraction patterns of the MAPbI_3_/P3CT-Na/ITO/glass samples fabricated with different drying times. The asymmetric curve indicates that the (110)-oriented peak and (002)-oriented peak overlapped.^55^ To separate the diffraction features of the two peaks, the asymmetric peaks are fitted with a two-Gaussian function. The fitting results are plotted in Fig. 6(b)–(d). The peak intensity values, the diffraction angles of peaks and the intensity ratio between the two peaks are listed in Table 2. The trend of the peak intensity values is inversely proportional to the trend of the (002)-peak position values, which is related to the lattice distortion of the MAPbI_3_ crystalline thin films. As shown in Fig. 6(c), the diffraction angle of the (002) peak is shorter than the others, which means that the crystal plane distance along the (002) direction is longer than the others when the drying time is 30 s. In a (110)-oriented MAPbI_3_ thin film, the (002) crystal plane is parallel to the inter-grain surface. In other words, the longer crystal plane distance along the (002) direction can be explained due to the tensile stress from the interfacial contact and the formation of merged grains. Besides, the intensity ratio of the (110)-peak and (002)-peak (*I*_110_/*I*_002_) is the highest value (14.58) when the drying time is 30 s. In other words, the crystal orientation of the merged MAPbI_3_ grains is also related to the surface properties of the P3CT-Na/ITO/glass substrate, thereby forming the (110) preferred MAPbI_3_ crystalline thin film.^56^Fig. 7 presents the top-excited PL spectra of the MAPbI_3_/P3CT-Na/ITO/glass samples fabricated with different drying times. The trend of the PL intensities is proportional to the trend of the *I*_110_/*I*_002_ ratio values (see Table 2). When the drying time is 30 s, the PL intensity from the top region of the MAPbI_3_ crystalline thin film corresponds to the highest value. When the drying time is reduced to 20 s, the relatively lower PL intensity can be explained as due to the higher defect density at the inter-gain boundaries of the MAPbI_3_ crystalline thin film. When the drying time is extended to 40 s, the relatively lower PL intensity can be explained as due to the higher surface defect density of the rough MAPbI_3_ crystalline thin film. Fig. 8 presents the absorbance spectra of the MAPbI_3_/P3CT-Na/ITO/glass samples fabricated with different drying times. In the near-infrared wavelength range from 800 nm to 1050 nm, the formation of ripples is due to the thin-film interference between interfaces. The larger amplitude of the interference ripple indicates the weaker light scattering from the surface due to the lower roughness of the MAPbI_3_ thin films.^57,58^ The trend of the ripple amplitudes is inversely proportional to the trend of the Ra values (see Fig. 5). Besides, the higher absorbance is due to the larger Ra when the drying time is 40 s. In general, the slope of the absorbance curve in the continuum absorption band is inversely proportional to the exciton binding energy of the MAPbI_3_ crystalline thin film.^59^ It is noted that the trend of the exciton binding energies is inversely proportional to the trends of the *J*_SC_ values and FF values (see Table 1). In other words, the higher PCE of the inverted perovskite photovoltaic cells is also related to the lower exciton binding energy of the MAPbI_3_ crystalline thin film due to the formation of merged and soft perovskite grains.

**Table 2:** Key features in the main XRD patterns, PL spectra and absorbance spectra of the MAPbI_3_/P3CT-Na/ITO/glass sample. MAPbI_3_ films are prepared with different drying times

Drying time	XRD peak intensity (counts)	2*θ* of the 002-peak (degree)	2*θ* of the 110-peak (degree)	Ratio of *I*_(110)_/*I*_(002)_	PL peak intensity (counts)	Slope of the absorbance curve (1/nm)
20 s	8141	14.072	14.086	1.36	48	−0.0097
30 s	6809	13.985	14.085	14.58	67	−0.0105
40 s	8141	14.070	14.090	1.72	38	−0.0088

**Fig. 9:**
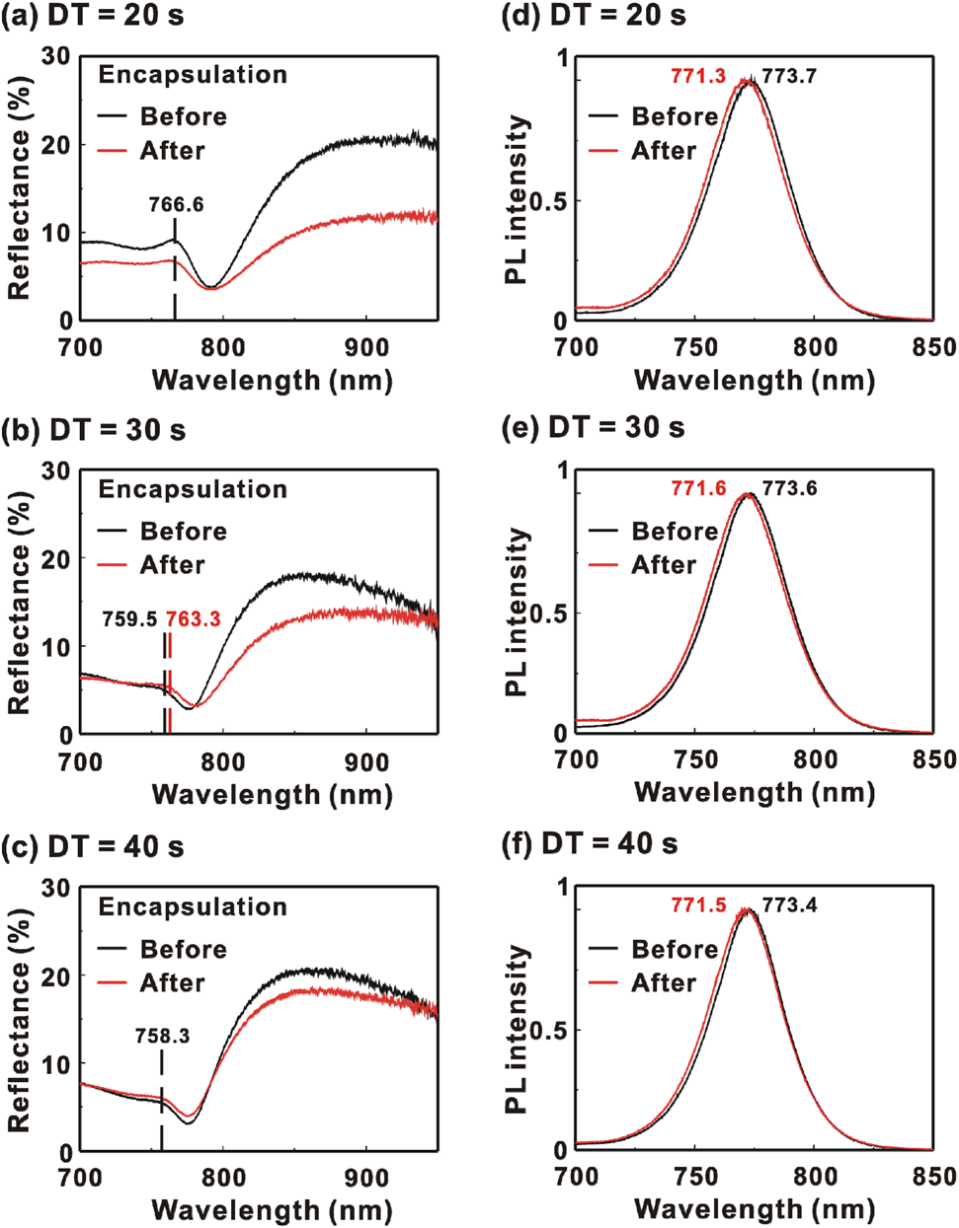
Reflectance and photoluminescence (PL) spectra of the MAPbI_3_/P3CT-Na/ITO/glass samples before and after encapsulation. MAPbI_3_ films are fabricated with different drying times (DTs). (a) Reflectance, DT = 20 s; (b) reflectance, DT = 30 s; (c) reflectance, DT = 40 s; (d) PL, DT = 20 s; (e) PL, DT = 30 s; (f) PL, DT = 40 s.

Fig. 9 presents the reflectance spectra and bottom-excited PL spectra of the MAPbI_3_/P3CT-Na/ITO/glass samples before and after the encapsulation process. The MAPbI_3_ crystalline thin films are fabricated with different drying times. The reflectance peak is related to the exciton transition of the MAPbI_3_ crystalline thin film. According to the material dispersion relation, a longer exciton transition wavelength results in a longer reflectance peak wavelength due to the higher refractive index.^60^ Therefore, the difference between the reflectance peak wavelength and the PL peak wavelength (Δ*λ* = *λ*_PL_ − *λ*_R_) is proportional to the Stokes shift (exciton binding energy). The reflectance peak wavelengths, PL peak wavelengths and Δ*λ* values of the MAPbI_3_/P3CT-Na/ITO/glass samples are listed in Table 3. Before encapsulation, the trend of the Δ*λ* (exciton binding energy) values is proportional to the trend of the grain sizes (see Fig. 5(d)–(f)), which means that the formation of merged and soft grains results in lattice distortion, thereby increasing the Δ*λ* (exciton binding energy) in the bottom region of the MAPbI_3_ crystalline thin film.^61^ In other words, the extended drying time results in a distorted lattice in the bottom region of the MAPbI_3_ crystalline thin film, thereby decreasing the atomic contact strength at the MAPbI_3_/P3CT-Na interface (see Fig. 5(d) and (e)). In the aspect of the device performance of un-encapsulated photovoltaic cells on the first day, the average *V*_OC_ value increases from 1.010 V to 1.017 V with the increase in the drying time from 20 s to 40 s, which can be explained due to the increased grain size (decreased potential loss). However, the average *J*_SC_ (FF) value has the highest value when the drying time is 30 s, which is due to the better contact quality at the MAPbI_3_/P3TC-Na interface and the lower exciton binding energy of the MAPbI_3_ thin film.

**Table 3:** Reflectance peak wavelength and PL peak wavelength of the MAPbI_3_/P3CT-Na/ITO/glass samples before encapsulation, after encapsulation and after 10 days. MAPbI_3_ films are fabricated with different drying times

Encapsulation	Drying time	Reflectance peak wavelength, *λ*_R_ (nm)	PL peak wavelength, *λ*_PL_ (nm)	Δ*λ* = *λ*_PL_ − *λ*_R_ (nm)
Before	20 s	766.6	773.7	7.1
Before	30 s	759.5	773.6	14.1
Before	40 s	758.3	773.4	15.1
After	20 s	766.6	771.3	4.7
After	30 s	763.3	771.6	8.3
After	40 s	758.3	771.5	13.2

After encapsulation, the decreased Δ*λ* values are 2.4, 5.8 and 1.9 nm when the drying times are 20, 30 and 40 s, respectively. It is noted that the trend of the decreased Δ*λ* (exciton binding energy) values is proportional to the trend of the increased *V*_OC_ values (see Table 1) after encapsulation. The decreased Δ*λ* (exciton binding energy) in the bottom region of the MAPbI_3_ crystalline thin film can be explained as due to the formation of shallow defects after the encapsulation process, which increases the effective Fermi level for electrons of the soft MAPbI_3_ crystalline thin film, thereby resulting in the higher *V*_OC_ of the inverted perovskite photovoltaic cells.

## Conclusions

In summary, the open-circuit voltage of the inverted perovskite crystalline photovoltaic cells can be largely increased from 1.010 V to 1.156 V *via* the formation of merged and soft CH_3_NH_3_PbI_3_ (MAPbI_3_) grains and the reduced exciton binding energy (increased carrier density), which are confirmed by analyzing the surface morphologies, cross-sectional images, crystal structures and excitonic properties of the MAPbI_3_/P3CT-Na/ITO/glass samples with and without encapsulation. It is noted that the photovoltaic cells maintain the average power conversion efficiency to be higher than 17.5% within 80 days when the MAPbI_3_ thin film is fabricated with an optimized drying time of 30 s.

## Conflicts of interest

There are no conflicts to declare.

## Supplementary Material

NA-005-D2NA00929C-s001
