# Supplementary material for: Effects of drying time on the formation of merged and soft MAPbI3 grains and their photovoltaic responses
Source: Nanoscale Adv. 2023 Mar 2;5(8):2190–8. doi: 10.1039/d2na00929c (PMC10089098; doi:10.1039/d2na00929c)
Supplement: NA-005-D2NA00929C-s001 [file NA-005-D2NA00929C-s001.pdf]

Electronic Supplementary Material (ESI) for Nanoscale Advances.
This journal is © The Royal Society of Chemistry 2023

Supporting information

Effects of drying time on the formation of merged and soft $MAPbI_3$ grains and their photovoltaic responses

Anjali Chandel[1,2,3], Qi Bin Ke[1,2,3], Shou-En Chiang[1,2,3], Hsin-Ming Cheng[4,*], Sheng Hsiung Chang[1,2,3,**]

[1]Department of Physics, Chung Yuan Christian University, Taoyuan 320314, Taiwan, ROC

[2]Research Center for Semiconductor Materials and Advanced Optics, Chung Yuan Christian University, Taoyuan 320314, Taiwan, ROC

[3]Center for Nanotechnology, Chung Yuan Christian University, Taoyuan, 320314, Taiwan, ROC

[4]Department of Photonics, National Cheng Kung University, Tainan 701, Taiwan, ROC

[*]smcheng.jemmy@gmail.com

[**]shchang@cycu.edu.tw

Corresponding authors: Hsin-Ming Cheng[*], Sheng Hsiung Chang[**]

Telephone: +886-3-2653208

Fax : 886-3-2653299

Fabrication and environment conditions

P3CT-Na/water solution (42 wt%) was spin-coated on top of the ITO/glass substrate at room temperatures. The environmental humidity was controlled to be lower than 50% RH. The spin rate and time of the P3CT-Na/water solution are 4000 rpm and 30 s, respectively.

$MAPbI_3$ precursor solution (1.5 M) was spin-coated on top of the P3CT-Na/ITO/glass sample at room temperatures in a nitrogen-filled glove box. A two step spin coating condition was used for the deposition of the $MAPbI_3$ thin film. In the first step, the spin rate and time are 400 rpm and 3 s, respectively. In the second step, the spin rate is 4000 rpm and the spin time is 29 s, 39 s or 49 s. During the spin coating process, a 180-μL CB was dropped at the 12th s. After the spin coating process, the sample was heated at 100 ℃ for 10 min, which formed $MAPbI_3$ crystalline thin film on top of the P3CT-Na/ITO/glass substrate.

PCBM/CB:BrB solution (2 wt%) was spin-coated on top of the $MAPbI_3$/P3CT-Na/ITO/glass sample at room temperature in a nitrogen-filled glove box. The spin rate and time are 1200 rpm and 30 s, respectively.

Ag film was deposited on top of the BCP:PCBM/$MAPbI_3$/P3CT-Na/ITO/glass sample by using the thermal evaporation method in a vacuum chamber. The pressure of the vacuum chamber is lower than 2×10^{-6} Torr. The deposition rate was lower than 0.1 nm/s.

Encapsulation process

The photovoltaic cells were encapsulated by using a sandwich structure as shown in **Figure S1**. The cells were placed on top of the hotplate with fixed a temperature at 55 °C. One glass plate and one parafilm were used to cover the cell under a fixed weight of 1 Kg. The thicknesses of glass plate and Parafilm are 0.7 mm and 0.13 mm, respectively. The time for encapsulation process is 1 minute.

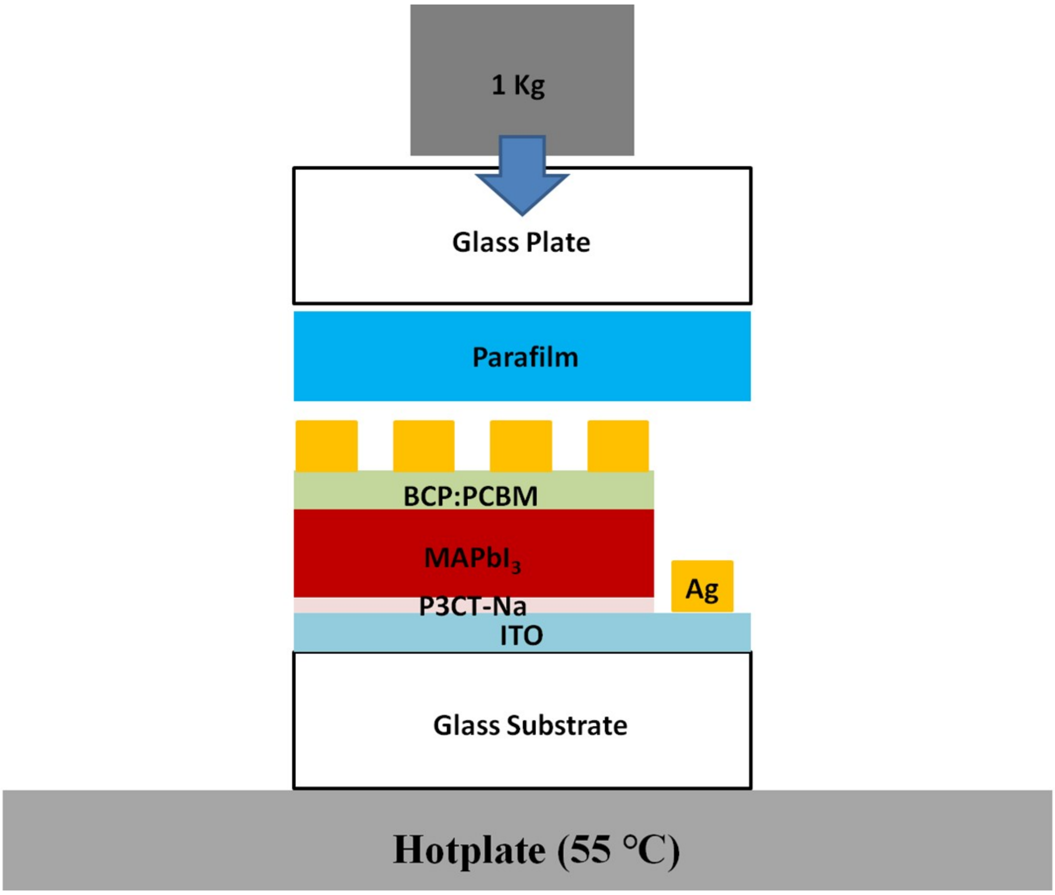

Fig. S1. Encapsulation of the photovoltaic cells.

Setups of the transmittance and reflectance spectrometer

Figure S2 presents two setups for the absorbance (transmittance) spectrum and reflectance spectrum, respectively**.** In **Figure S2 (a)**, the transmittance (T) of the sample can be measured when flipping the mirror down. The absorbance (A) can be calculated by using the equation (A=-log(T)). In **Figure S2 (b)**, the reflectance of the sample can be measured when flipping the mirror up. The mirror on the motorized linear stage is used as the reference of the reflectance spectrum.

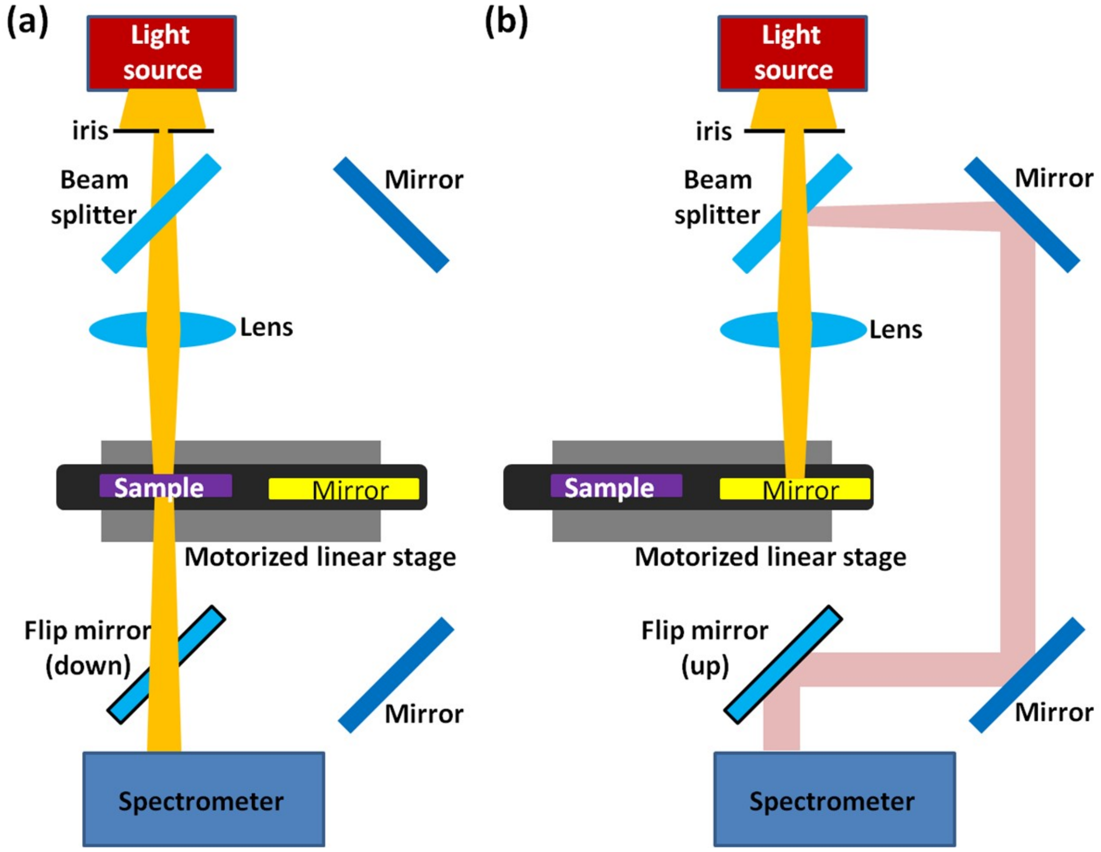

Fig. S2. (a) Setup for absorbance (transmittance) spectrum. (b) Setup for reflectance spectrum.

JV curve and V_{OC} hysteresis of the encapsulated $MAPbI_3$ solar cells

Figure S3 (a), **(b)** and **(c)** present the forward and backward J-V curves of the encapsulated $MAPbI_3$ solar cells fabricated with the different drying times. The drying time-dependent V_{OC} hysteresis is plotted in **Figure S3 (d)**.

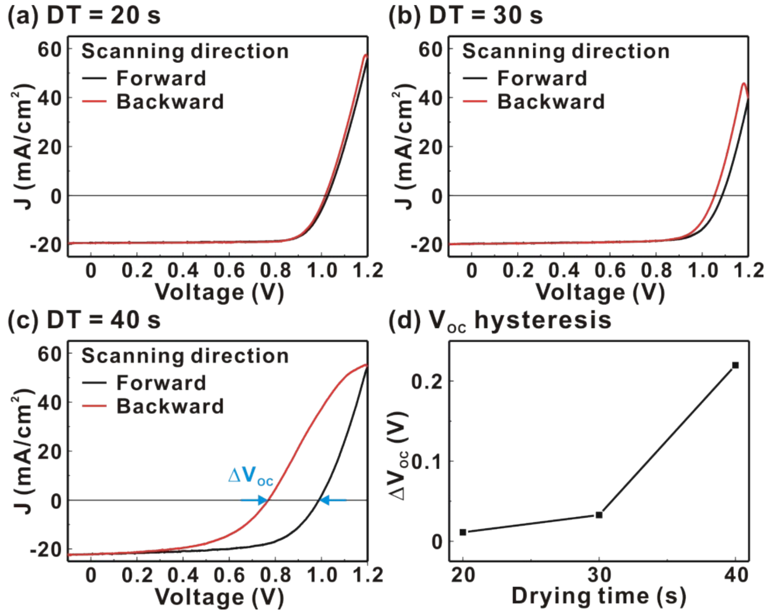

Fig. S3. Forward and backward current density-voltage curves of the encapsulated MAPbI3 solar cells fabricated with the different drying times (DTs). (a) DT = 20 s; (b) DT = 30 s; (c) DT = 40 s. (d) Drying time dependent V_{OC} hysteresis.

Experimental data and fitting curves of day-dependent V_{OC} and FF values

Figure S4 presents the experimental data and fitting curves of the V_{OC} and FF values when the encapsulated $MAPbI_3$ solar cells are fabricated with the different drying times from 20 s to 40 s.

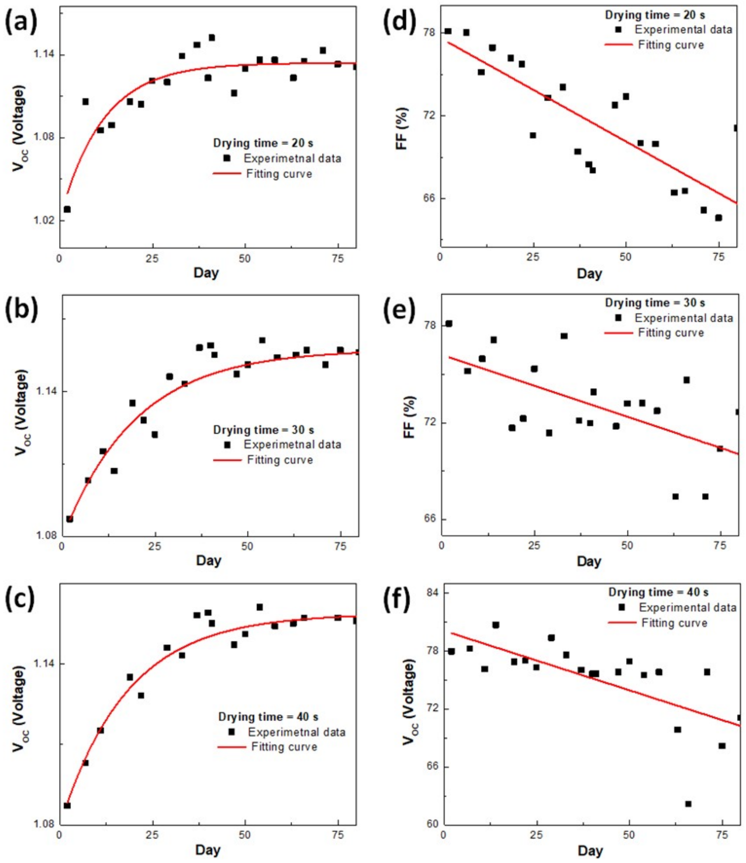

Fig. S4. Experimental data and fitting curves of day-dependent V_{OC} and FF values. (a) V_{OC}, drying time = 20 s; (b) V_{OC}, drying time = 30 s; (c) V_{OC}, drying time = 40 s; (d) FF, drying time = 20 s; (e) FF, drying time = 30 s; (f) FF, drying time = 40 s.

Incident photon-to-current conversion efficiency (IPCE) spectra

Figure S5 presents the IPCE spectra and the integrated current density curves of the encapsulated perovskite photovoltaic cells measured on the 407th day. **Equation S1** is used to calculate the IPCE.

$$\mathrm{IPCE}(\lambda)=\frac{J_{\mathrm{ph}}\times 1240}{I_{light}\times\lambda}, \qquad \text{(S1)}$$

where J_{ph} is the measured photocurrent density, I_{light} is the intensity of incident photons, and λ is the wavelength of incident photons. In the measurements, the light intensity is fixed at 4 mW/cm^2. The wavelength is varied from 450 nm to 850 nm.

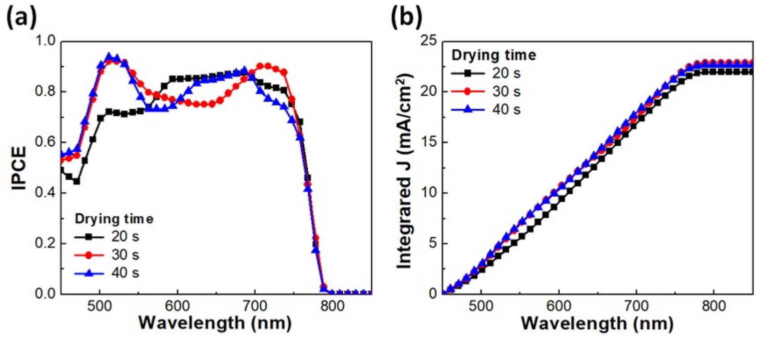

Fig. S5. (a) IPCE spectra of the encapsulated perovskite photovoltaic cells. (b) Integrated current density curves of the encapsulated perovskite photovoltaic cells.
